# Exploring diet-induced ketosis with exogenous ketone supplementation as a potential intervention in post-traumatic stress disorder: a feasibility study

**DOI:** 10.3389/fnut.2024.1406366

**Published:** 2024-10-31

**Authors:** Maria G. P. Edwards, Tobias Furuholmen-Jenssen, Erik Ganesh Iyer Søegaard, Suraj Bahadur Thapa, Jens R. Andersen

**Affiliations:** ^1^Department of Nutrition, Exercise and Sports, University of Copenhagen, Copenhagen, Denmark; ^2^Division of Mental Health and Addiction, Oslo University Hospital, Oslo, Norway; ^3^Division of Mental Health and Addiction, Institute of Clinical Medicine, University of Oslo, Oslo, Norway

**Keywords:** post-traumatic stress disorder (PTSD), ketogenic diet (KD), ketosis, ketogenic metabolic therapy (KMT), ***β***-hydroxybutyrate (BHB), exogenous ketones, ketone salts (KS)

## Abstract

**Background:**

Post-Traumatic Stress Disorder (PTSD) is a severe and pervasive mental disorder, and patients experience numerous distressing symptoms and impairments that significantly impact their lives. In addition to being a mental disorder, PTSD is strongly associated with a wide range of metabolic abnormalities that affect the entire body. Existing treatment options of psychotherapy and medications are often ineffective. Exploring other potential treatments is necessitated. The ketogenic diet has shown potential as a metabolic therapy in certain neurological and mental disorders and is a promising intervention in the treatment of PTSD.

**Aim:**

This study aimed to examine if a 4-week ketogenic diet intervention supplemented with exogenous ketones (KD-KS) was feasible in adult patients with PTSD, to what extent it was possible to recruit patients, attain and maintain ketosis (plasma concentration of *β*-hydroxybutyrate (BHB) ≥ 0.5 mmol/L), the occurrence of serious adverse reactions and adverse reactions to KD-KS, and acceptance of treatment. Our exploratory aims were changes in PTSD symptoms and health-related quality of life (QoL) from baseline to 4 weeks.

**Methods:**

Patients 18 ≤ 65 years old, diagnosed with PTSD, and receiving outpatient treatment for PTSD at Southern Oslo District Psychiatric Centre (DPC), Oslo University Hospital, Oslo, Norway, were included. The intervention consisted of a ketogenic diet supplemented with *β*-hydroxybutyrate salt to obtain ketosis. PTSD symptoms were measured with the PTSD Checklist for DSM-5 (PCL-5) and QoL was measured with the RAND 36-Item Health Survey 1.0.

**Results:**

During a 21-week inclusion period, three of four eligible patients (75% [95% CI: 30 to 95%]) were included. Two patients (67% [95% CI: 21 to 94%]) completed the 4-week intervention and one patient (33% [95% CI: 6 to 79%]) completed 2 weeks of intervention before discontinuing. Ketosis was achieved on day 1 in one patient, and on day 2 in two patients, and was maintained in 87% of the intervention. There were no serious adverse reactions. Adverse reactions were reported in a total of 70% of intervention days, the most frequent being headache followed by fatigue. The participant-perceived degree of adverse reactions was low to moderate. The treatment was accepted by patients on all intervention days. PCL-5 decreased by 20 points (70 to 50) in patient 1 and by 10 points (50 to 40) in patient 2, from baseline to 4 weeks, which is a reliable and clinically meaningful improvement. QoL improved in six of eight RAND-36 subscales in patient 1 and three of eight in patient 2. Patient 3 did not complete assessments after week 2.

**Conclusion:**

To the best of our knowledge, this feasibility study is the first study examining a ketogenic diet intervention in patients with PTSD. Three of four predefined feasibility criteria were achieved. Ketosis was attained fast and maintained, patients were compliant and there were clinically meaningful improvements in PTSD symptoms and QoL. Despite the small sample size, the knowledge obtained in this study is important for the planning of future studies with ketogenic diet interventions in this patient group. It is a first step for potential dietary and metabolic therapies in PTSD. Further feasibility and pilot studies with larger sample sizes are needed to determine feasibility and safety before planning future randomised controlled trials investigating an effect.

**Clinical trial registration:**

https://ClinicalTrials.gov, identifier NCT05415982.

## Introduction

Post-Traumatic Stress Disorder (PTSD) is associated with long-lasting changes and dysfunction in many of the body’s biological systems, predisposing both somatic and mental disorders, causing disability, and limiting the quality of life (1–5). Most individuals with trauma disorders have several comorbidities such as anxiety, psychotic, depressive, psychosomatic, eating, and conduct disorders, changes in personality, cognitive difficulties and sexual dysfunction. The stress sustained from PTSD may lead to somatic diseases (cutaneous, digestive, cardiovascular, endocrine and auto-immune) (6). This calls for and necessitates a holistic approach to the treatment of affected trauma patients. PTSD is characterized by a severe and prolonged reaction, often chronic, to a distressing event of an exceptionally threatening or catastrophic nature. Repeated exposure to traumatic events increases the risk of developing PTSD (6, 7). Complex PTSD is a relatively new diagnosis that was included in the International Classification of Diseases, 11th Revision (ICD-11) in June 2018 (8, 9). It is a subtype of PTSD that includes additional symptoms related to prolonged trauma exposure, such as chronic and pervasive disturbances in emotion regulation, identity, and relationships (10). A cross-sectional study on PTSD in Norway showed a lifetime prevalence of 4.3% for women and 1.4% for men, with the average duration of the disorder being nine and 17 years, respectively (11). It is estimated that approximately 1–2% of the population has PTSD at any given time in Norway, which is roughly consistent with European levels (12, 13). Studies suggest that PTSD is underdiagnosed because trauma is often not inquired about, leading to misdiagnosis and mistreatment (14, 15). Studies of individuals at high risk of developing PTSD, i.e., populations with high exposure to trauma, show higher rates (16). In the Norwegian Armed Forces’ Afghanistan Report 2020, it is reported that 2.9% of Norwegian Afghanistan veterans who have been exposed to one or more traumatic events have PTSD (17), and 31% of female rape victims were diagnosed with PTSD shortly after the assaults during the war in Croatia and Bosnia-Herzegovina (1991–1995) (18). Similar figures are seen today in Norway, as Norwegian Health Informatics states that just over 30% of rape victims develop PTSD (7). Up to 50% may develop PTSD if the event is not processed, e.g., with trauma therapy (19). A Swedish study showed that 79% of refugees in Sweden who had been subjected to highly traumatic events such as war, torture, and captivity had PTSD (20).

Several neurobiological systems are altered in patients with PTSD (1, 21). Changes have been described in the neuroendocrine system, in the autonomic nervous system with increased sympathetic activation, metabolic changes, increase in inflammation, and imbalance in neurotransmitters (1, 21, 22). Hyperarousal is a cardinal symptom of PTSD. This happens through an increase in brain activity in the limbic and neuroendocrine systems, which leads to increased vigilance and alertness. This heightened activation is also thought to maintain the disorder (1, 2).

PTSD is associated with a wide range of mental, social, and physical disorders (1, 4, 6). PTSD patients are more likely to have hyperglycaemia, hypertriglyceridaemia, high levels of low-density lipoprotein (LDL) and low levels of high-density lipoprotein (HDL) cholesterol, and high blood pressure (3, 23). All are components of metabolic syndrome and factors associated with increased risk of type 2 diabetes and cardiovascular disease, indicating that metabolism outside the brain is also affected (24, 25). Epidemiological data suggest that PTSD increases the risk of developing metabolic syndrome, cardiovascular disease, and premature death (4, 5, 26). In addition to being a mental disorder, PTSD can be seen as a metabolic disorder that affects the entire body (4, 5, 26). The changes result from chronic elevated stress, altered lifestyle, and medications. Targeted therapy aimed at some of the many underlying biological systems that are altered, such as disrupted Hypothalamus-Pituitary–Adrenal axis (HPA axis), high and sustained activation of the sympathetic nervous system, and inflammation, can potentially help with symptomatic improvement for the individuals affected (27, 28).

Existing treatments consist of various forms of psychotherapy and exposure therapy, largely aimed at fear extinction and memory reconsolidation (29, 30). In some cases, medications such as anxiolytics and antidepressants are given, which may often be more targeted towards sequelae or comorbidities that often occur concurrently with PTSD. Several individuals with PTSD struggle to benefit effectively from treatment due to strong avoidance symptoms and the inability to complete overwhelming exposure. Patients may become hyperaroused during treatment and end up outside the window of tolerance where they can process traumas and consolidate fragmented memories. Around 30% drop out of treatment due to the strain they experience from hyperarousal (29). Many patients are treatment-resistant to the established treatment options available today and may go years without getting better (30). The lack of effective treatment options, especially for the most severely affected trauma patients, necessitates exploring other potential treatments. One possible approach could be a ketogenic diet (KD). The use of lifestyle interventions in the treatment of chronic diseases is attractive due to their availability, possibly fewer side effects, and the potential for reduced medical costs (31, 32).

Physiological ketosis is a metabolic state achieved through fasting, starvation, prolonged intensive exercise, or by following a KD (33–35). A KD is a high-fat, moderate protein, very low carbohydrate diet that induces metabolic changes resembling those seen in a fasting state (33, 35). By restricting carbohydrates, fat oxidation increases in several tissues, and fatty acids are metabolized into ketone bodies (KB) in the liver, which are used as alternative cerebral energy substrates to glucose. KB, such as *β*-hydroxybutyrate (BHB) and acetoacetate (AcAc), can supply up to 60–70% of the basal cerebral energy requirements (35–38). BHB is also a signalling metabolite that affects epigenetic gene regulation and cellular function and has important neuroprotective effects (39–42). The definition of physiological ketosis is an increased serum concentration of KB ≥ 0.5 mmol/L (35, 43, 44).

Exogenous ketosis can be achieved by consuming exogenous ketone supplements in the form of ketone salts or esters, and ketone salts can also be infused intravenously. Ketone salts consist of BHB bound to minerals like sodium, potassium, or calcium, whereas in ketone esters, BHB and/or AcAc is ester bonded to an alcohol (45). Exogenous ketones can generate rapid, mild to moderate therapeutic ketosis (approximately 1–7 mmol/L) (46–52) and can be combined with a KD to elevate ketone levels and to maintain ketosis if there is a lack of compliance with the KD. For patients not able to, or not wishing to adhere to a KD, exogenous ketones can be added to a standard diet or a less strict moderate to low-carb diet, to induce and maintain exogenous ketosis. To sustain ketosis, exogenous ketones must be administrated several times a day, depending on the dosage and type of exogenous ketone supplement. Ketone esters are prone to elevate blood BHB (b-BHB) more and faster than ketone salts. Ketone salts, and sometimes ketone esters, are often racemic mixtures of the two optical isoforms of BHB, D-BHB and L-BHB, and the metabolism and function of L-BHB are poorly understood (47). D-BHB is the predominant circulating KB and is better oxidized than L-BHB, which only accounts for 2–3% of endogenous BHB production in the fasted state (53, 54). This has led to L-BHB being thought of as not important, which might not be the case (54–56). L-BHB is metabolized more slowly, suggesting that a racemic mixture sustains ketosis for longer (57, 58). Commercial ketone meters and standard laboratory analysis only detect D-BHB, and most do not test for AcAc (53).

KD is an established and effective treatment for refractory epilepsy used since the 1920s, and fasting has been known to reduce seizures for centuries (33, 59–61). To exhibit the anticonvulsant effect seen in epilepsy, KD must suppress excitation in neurons, regardless of underlying mechanisms, and a bidirectional relation between epilepsy and mood disorders is hypothesised (62–64). There are few studies on KD in mental disorders, but some have shown promise in major depressive disorder, bipolar disorder, schizoaffective disorder, schizophrenia, autism spectrum disorder, anorexia nervosa and substance/alcohol use disorder (65–83), and more studies are on their way (84, 85). There is also a therapeutic potential of exogenous ketone supplements in mental disorders (51, 86, 87). It is conceivable that KD raises the threshold for neuronal excitation and contributes to synaptic stability in PTSD, as seen in other conditions (88, 89). Currently the authors are not aware of any previous studies on KD as an intervention for PTSD, yet the hyperexcitability hypothesis may be equally relevant in this condition (90). KD may thus reduce symptom expression in PTSD patients.

The mechanisms of action of KD are not fully understood. Some assumed important components include a decrease in neuroinflammation, reactive oxygen species (ROS) and redox stress, improved energy metabolism/mitochondrial function, membrane properties, direct stimulation of transcription factors and epigenetic changes, ion channels, and maintenance of membrane potential (39, 70, 91). From previous studies, KD seems to be effective in treating dyslipidaemia and reducing systemic inflammation (35, 92–95). This speaks to KD as a promising candidate in the treatment of PTSD, which shares several of the same metabolic abnormalities (1). KD has shown potential as metabolic therapy for a range of metabolic, neurological, and mental disorders, and the mechanisms are now investigated in, e.g., brain injury, migraine, mental illness, Alzheimer’s disease, cancer, and diabetes (38, 70, 88, 96–104). Metabolism is a key feature of neurological health and stability, and its role in the treatment of mental disorders has started to receive much attention in the young research field of Metabolic Psychiatry (65, 77, 85, 105–107).

Despite the growing body of evidence supporting the beneficial effects of the KD on the brain, its application in the context of PTSD remains largely unexplored. Based on results from studies in other mental disorders, it was hypothesised that a KD supplemented with BHB salt (KD-KS) may alleviate symptom expression in PTSD patients and no previous studies have been published testing this hypothesis. Therefore, we designed a study to investigate if this intervention is feasible in adult PTSD patients, during a 4-week intervention. The primary aims were to examine to what extent it was possible to recruit patients, attain and maintain ketosis, the occurrence of serious adverse reactions (SARs) and adverse reactions (ARs) to the KD-KS, and acceptance of the intervention. Our exploratory aims were changes in PTSD symptoms and health-related quality of life from baseline to 4 weeks.

## Materials and methods

This single-site, non-randomised, open-label, single group feasibility study was conducted over 6 months on outpatients with PTSD, receiving treatment at Southern Oslo District Psychiatric Centre (DPC), Oslo University Hospital, Oslo, Norway. This study is reported following the Consolidated Standards of Reporting Trials (*CONSORT*) statement (Supplementary Table S3) (108). Ethics approval was granted by the Regional Committees for Medical and Health Research Ethics South East Norway (REK) (455897) and the study was registered at https://clinicaltrials.gov (Identifier: NCT05415982) on 23 March 2022. The study was conducted following the principles of the Declaration of Helsinki (109).

### Participants

Patients 18 ≤ 65 years old, diagnosed with PTSD, speaking a Scandinavian language, and receiving outpatient treatment for PTSD at DPC, were included. Exclusion criteria were if KD was contraindicated (110) (Supplementary Table S1), body mass index (BMI) < 18, dysregulated diabetes mellitus, treatment with medication for elevated plasma triglycerides, and pancreas-, kidney-, or liver disorders. Following the study entry, baseline measurements were taken including psychological tests, blood samples, and weight measurements (Supplementary Table S2). Energy requirements were estimated from the Harris-Benedict equation corrected for stress and activity factor (111). Ketogenic ready meals (Natural Ketosis, Edinburgh, United Kingdom), ready-to-drink/semi-solid ketogenic formulas (K.Flo^®^/K.Yo^®^, Vitaflo International Ltd., Liverpool, United Kingdom), low-carb food products that facilitated the intervention, an exogenous ketone supplement (Ketostart^®^, Audacious Nutrition LLC, Tampa, Florida, United States), as well as a blood ketone/glucose meter and test strips (Keto-Mojo Europe B.V., Amsterdam, Netherlands) were provided. Participants were trained to perform blood ketone/glucose measurements by finger-prick testing and received instructions on how to register their nutrition intake. They were given a self-assessment form to register serious adverse events (SAEs), adverse events (AEs), SARs, ARs, acceptance of intervention and nutrition intake. Nutrition and fluids were registered daily by the participants and calculated by the investigator using a Danish internet-based software “Vitakost” for nutritional contents (112). The investigator saw the participants weekly at DPC for an interview, in conjunction with blood sampling, and maintained regular contact, at least several times a week, to monitor their progress and the intervention.

### Nutritional intervention

The ketogenic food products provided were intended as full nutrition during the intervention, to ensure a macronutrient composition leading to ketosis. Participants were encouraged to predominantly consume the products but also had the option to prepare their own ketogenic meals with guidance from the investigator. Six different ketogenic ready meals (Natural Ketosis, Edinburgh, United Kingdom) were provided for lunch and dinner, and due to import regulations between the United Kingdom and Norway, all meals were vegan. Participants were encouraged to add additional animal or plant protein to the meals if wanted. As breakfast and meal replacement K.Flo^®^ and K.Yo^®^ (Vitaflo International Ltd., Liverpool, United Kingdom) were used. K.Flo^®^ is a ready-to-drink nutritionally complete, 4:1 ratio, ketogenic formulation in vanilla flavour, consisting of 14.7 g fat, 1.6 g carbohydrate, and 3.4 g protein per 100 mL. K.Yo^®^ is a nutritionally complete, 3:1 ratio, semi-solid ketogenic formulation in vanilla or chocolate flavour, consisting of 30 g fat, 1.5–2.0 g carbohydrate, and 8 g protein per 100 g. To increase levels of b-BHB and to keep participants in ketosis even if not 100% compliant with the intervention, the exogenous ketone supplement Ketostart^®^ (Audacious Nutrition LLC, Tampa, Florida, United States), was used. Ketostart^®^ is a BHB salt (racemic mixture containing D-BHB and L-BHB) in powder form with tropical flavour, consisting of 10 g BHB per serving (17 g). One serving Ketostart^®^ was mixed with water and consumed throughout the day. Patients were encouraged to consume sufficient salt and electrolytes during the intervention to meet increased demand on a KD.

### Feasibility outcomes

The primary objective of this trial was to assess feasibility. The intervention was considered to be feasible if all of the criteria in Table 1 were attained. Limits to determine feasibility were estimated from the results of previous trials (113–117).

**Table 1:** Feasibility outcomes.

Outcomes	Measures	Limit to be feasible
Recruitment of patients	Percent of included patients to eligible patients	≥60% of eligible patients included (114, 115)
Maintaining ketosis^a^	Percent of days in ketosis^a^ since ketosis^a^ was attained	≥75% of days in ketosis^a^ since ketosis^a^ was attained (113)
Occurrence of serious adverse reactions and adverse reactions to the ketogenic diet supplemented with BHB salt	Percent of intervention days with serious adverse reactions and adverse reactions	≤5% of intervention days with serious adverse reactions in 100% of patients ≤30% of intervention days with adverse reactions in ≥75% of patients (116, 117)
Acceptance of treatment	Yes/No, percent of intervention days	≥75% of patients accepting the treatment in ≥75% of intervention days (113)

a Ketosis was defined as a mean value of the three daily blood *β*-hydroxybutyrate measurements ≥ 0.5 mmol/L.

#### Maintaining ketosis

To assess the level of ketosis and fluctuations in blood glucose (BG), b-BHB and BG were monitored three times daily by the participants: morning, mid-day, and in the evening. It was encouraged that the participants take the measurements before meals and around the same time each day. b-BHB and BG were sampled by a finger-pricker and GKI-Bluetooth Blood Glucose & Ketone Meter^®^ (Keto-Mojo Europe B.V., Amsterdam, Netherlands), a device that measures both b-BHB (D-BHB) and BG with two different test strips. The meter uploaded the data via Bluetooth to MyMojoHealth app on the participants’ mobile phones and the investigator followed the data in real-time via the MyMojoHealth webpage for practitioners. This way the investigator could intervene if measurements were missed or if the data showed incompliance to the KD-KS. A day in ketosis was defined as a mean value of the three daily b-BHB measurements ≥0.5 mmol/L.

#### Occurrence of serious adverse reactions and adverse reactions to the ketogenic diet supplemented with *β*-hydroxybutyrate salt

Serious adverse events (SAEs), adverse events (AEs), serious adverse reactions (SARs), adverse reactions (ARs), and suspected unexpected serious adverse reactions (SUSARs) were monitored during the trial and reported according to ICH E6 Good Clinical Practice guidelines (118). SAEs were defined as an adverse event that results in death, is life-threatening, requires hospitalisation or prolongation of existing hospitalisation, results in persistent or significant disability or incapacity or requires intervention to prevent permanent impairment or damage, whether considered related to the trial intervention or not. AEs were defined as any untoward medical occurrence in a patient that does not necessarily have a causal relationship with the intervention. SARs and ARs were defined as any harmful and undesirable reaction with a direct causal relationship to the intervention, serious or not considered serious (118). SAEs, AEs, SARs, ARs, and participant-perceived degree of ARs (5-point Likert scale (0-4), 0: no adverse reaction, 4: strong adverse reaction) were documented daily by the participants on the self-assessment form and assessed by the investigator.

##### Biochemical analysis for safety

Venous plasma samples were analysed weekly (triglycerides, total cholesterol, LDL cholesterol, HDL cholesterol, sodium, potassium, magnesium, phosphate, C-reactive protein (CRP), metanephrine, normetanephrine) or every 2 weeks (alanine aminotransferase (ALAT), alkaline phosphate (ALP), bilirubin, creatinine) to assess safety. Haemoglobin A1c (HbA1c) was analysed at inclusion and completion/exclusion. All blood samples were taken postprandial and not in a fasted state, as the participants had their first meal before blood samples were taken at DPC.

#### Acceptance of treatment

Acceptance of treatment was documented daily by the participants on the self-assessment form and was defined as the patients being compliant with the dietary intervention and performing the daily measurements and registrations (yes/no, daily). The participants also filled out two separate 5-point Likert scales (0–4), one for the dietary intervention and one for daily measurements/registrations (0: not demanding, 1: slightly demanding, 2: moderately demanding, 3: quite demanding, 4: very demanding).

### Exploratory outcomes

#### Assessment of severity of PTSD symptoms and measure of health-related quality of life

Changes in PTSD symptoms from baseline to 4 weeks were assessed using the PTSD Checklist for DSM-5 (PCL-5) (119). PCL-5 is a 5-point scale with 20 questions. Each question is answered based on the frequency of experiencing a particular symptom, ranging from 0 (not at all) to 4 (very often). The minimum value is 0 and the maximum value is 80, the lower the score the better.

Health-related quality of life was assessed using the RAND 36-Item Health Survey 1.0 (RAND-36) (120) at baseline and after 4 weeks. The score provided represents a percentage of the total possible score, ranging from 0 to 100%. Higher scores are better, and 0 is worse. RAND-36 consists of 36 questions covering eight subscales, each scored from 0 to 100%: physical function, role limitations due to physical health, role limitations due to emotional problems, pain, general health perception, energy and fatigue, social function, and mental health.

#### Weight

Patients weighed themselves at home on a digital bathroom scale at baseline and weekly for 4 weeks. The weighing was in a standardised manner on the same scale in the morning, wearing light clothing, before consuming food and drinks, and after using the toilet. The patients sent pictures of the weight on the scale to the investigator.

### Statistical analysis

We aimed in this study to include 10 patients. As this is the first feasibility study with a KD intervention in patients with PTSD, we followed the recommendations of Steven A. Julious, as a formal power calculation was not feasible (121). No formal statistical comparison was carried out due to the low sample size.

## Results

### Feasibility outcomes

#### Recruitment of patients

**Figure 1:**
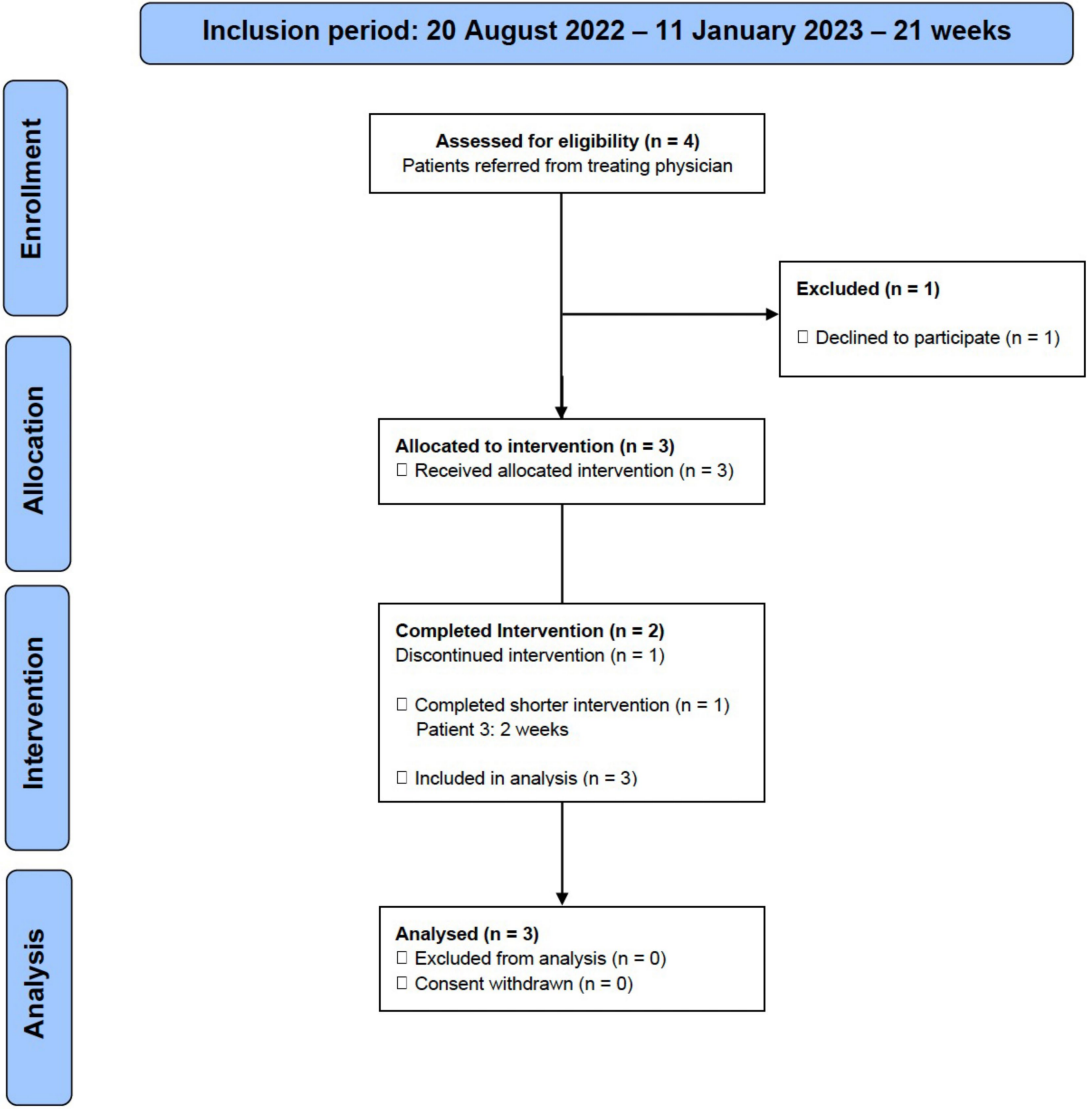
Flow diagram describing the inclusion and exclusion of patients in the study.

Four patients were found eligible during a 21-week inclusion period, and referred by the treating physician to the investigator, for further information about the study. It was possible to include three of four (75% [95% CI: 30 to 95%]) eligible patients. One patient found the intervention too demanding and declined to participate. Two patients (67% [95% CI: 21 to 94%]) completed the 4-week intervention. One patient (33% [95% CI: 6 to 79%]) completed 2 weeks of the intervention before discontinuing (Figure 1). The reason stated was that the intervention was too demanding, and the patient did not like the food. The patient felt unwell (did not specify how) and exhausted from the measurements/registrations and cooking. At baseline PCL-5 was 59 ± 10 (mean ± SD) and current comorbidities were chronic pain syndrome (fibromyalgia) (*n* = 3), chronic fatigue (*n* = 2), chronic headache (*n* = 2), bipolar disorder (*n* = 1), prediabetes (*n* = 1) and metabolic syndrome (*n* = 1). The baseline characteristics of the patients are summarised in Table 2.

**Table 2:** Patients’ baseline characteristics.

Variable	Intervention group (*n* = 3)
Age (years)	49 ± 4
Gender (Male/Female)	(0/3)
Weight	80.03 ± 15.08
Body mass index (BMI)	28.93 ± 5.49
**Diagnosis**	
Post-Traumatic Stress Disorder (PTSD)	3
**PTSD symptoms**	
PTSD Checklist for DSM-5 (PCL-5) (0–80 points)	59 ± 10
**Health-Related Quality of Life (RAND-36) (0–100% score)**	
Role limitations due to physical health	20 ± 18.03
Role limitations due to emotional problems	0 ± 0
Energy	6.67 ± 5.77
Mental health	34.67 ± 26.63
Social functioning	16.67 ± 7.22
Pain	15 ± 12.99
General health	15 ± 13.23
Physical function	62.5 ± 3.54
**Biochemistry**	
HbA1c (%)	33.33 ± 4.51
Alanine aminotransferase (ALAT) (U/L)	32.33 ± 25.15
Alkaline Phosphatase (U/L)	79 ± 25.06
Bilirubin (μmol/L)	6.33 ± 2.08
Triglycerides (mmol/L)	2.33 ± 1.99
Total cholesterol (mmol/L)	6.13 ± 1.62
Low-density lipoprotein (LDL) (mmol/L)	4.03 ± 1.49
High-density lipoprotein (HDL) (mmol/L)	1.47 ± 0.49
C-Reactive Protein (CRP) (mg/L)	5.20 ± 4.03
Metanephrine (nmol/L)	0.18 ± 0.06
Normetanephrine (nmol/L)	0.43 ± 0.04
**Current comorbidity**	
Prediabetes	1
Metabolic syndrome	1
Fatigue	2
Pain syndrome (fibromyalgia)	3
Headache	2
Bipolar disorder type 2	1

Data is presented as number of patients or mean ± SD.

#### Maintaining ketosis

**Figure 2:**
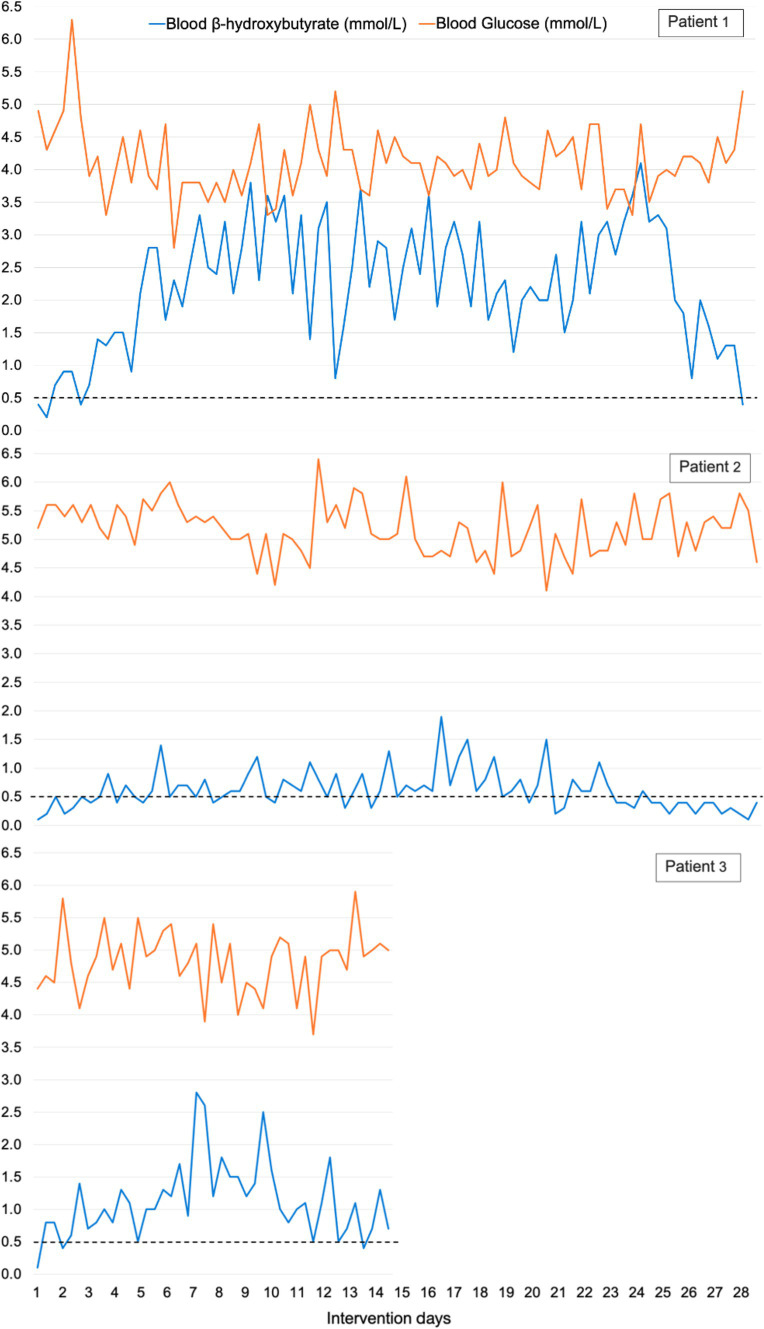
Blood *β*-hydroxybutyrate and blood glucose levels (mmol/L) in all patients. The three daily measurements are included and not daily mean values. Two of 126 (2%) of total measurements were missed, one blood *β*-hydroxybutyrate and one blood glucose measurement by patient 2.

**Figure 3:**
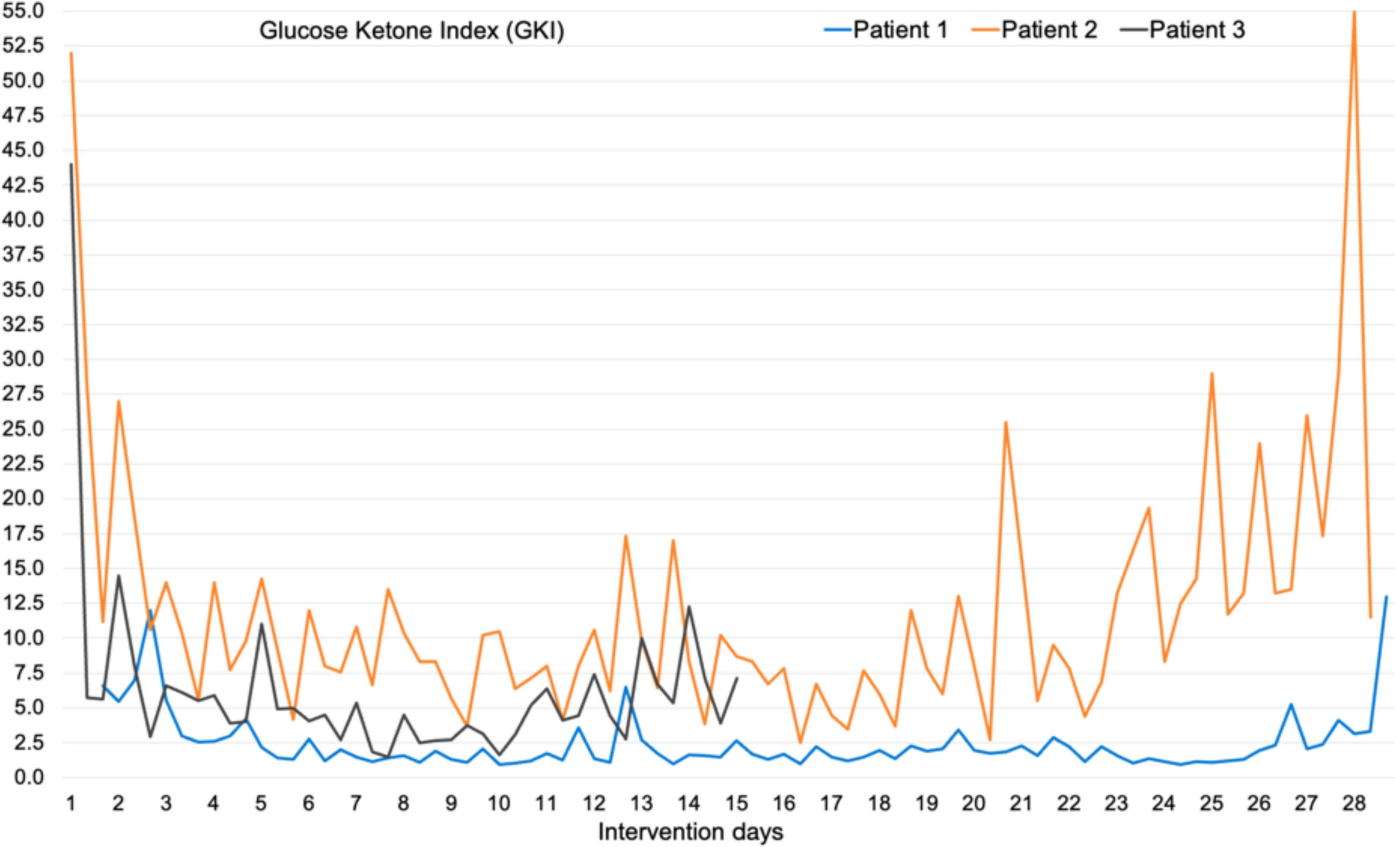
Glucose Ketose Index (GKI), the ratio of blood glucose to blood *β*-hydroxybutyrate, in all three patients. Calculated from all three daily measurements.

All patients attained at least one b-BHB measurement ≥0.5 mmol/L on the first day of intervention. The mean value of the three daily b-BHB measurements (a day in ketosis) were ≥ 0.5 mmol/L on day 2 in patients 1 and 2, and on day 1 in patient 3. Ketosis was maintained in 87% of the intervention. In 96% (27 of 28 days) in patient 1, 71% (20 of 28 days) in patient 2, and in 100% (14 of 14 days) in patient 3. Diagrams of all daily b-BHB and BG measurements throughout the intervention per patient, are found in Figure 2. Out of the planned three daily b-BHB and BG measurements, only 2% (2 of 126) of total measurements were missed (one b-BHB and one BG measurement by patient 2). The ratio of BG to b-BHB, Glucose Ketose Index (GKI) calculated from the three daily b-BHB and BG measurements are seen in Figure 3.

#### Occurrence of serious adverse reactions and adverse reactions to the ketogenic diet supplemented with *β*-hydroxybutyrate salt

There were no SAEs or AEs, and no observed SARs or SUSARs. All patients experienced ARs and in total there were 70% of intervention days with ARs, many of which occurred on the same days. The most frequent adverse reaction was headache followed by fatigue (Table 3). The participant-perceived degree of ARs was low in most ARs, the strongest perceived ARs were headache and fatigue (Table 3). The ARs did not affect the acceptance of treatment.

**Table 3:** Number and percent of days with adverse reactions in all patients and participant-perceived degree of adverse reactions measured with a daily self-assessed 5-point Likert scale (0: no adverse reaction, 4: strong adverse reaction).

Patient	Intervention days	Diarrhoea	Constipation	Nausea	Stomachache	Headache	Dizziness	Fatigue
1	28	1 (4%)	5 (18%)	14 (50%)	6 (21%)	8 (29%)	12 (43%)	5 (18%)
2	28	3 (11%)	18 (64%)	2 (7%)	3 (11%)	27 (96%)	0 (0%)	26 (93%)
3	14	1 (4%)	4 (29%)	4 (29%)	14 (100%)	14 (100%)	8 (57%)	14 (100%)

Participant-perceived degree of adverse reactions (0: no adverse reaction, 4: strong adverse reaction)								
1	28	0.11 ± 0.58 (0)	0.44 ± 1.09 (0)	1.5 ± 1.73 (0.5)	0.35 ± 0.75 (0)	0.59 ± 0.97 (0)	1.3 ± 1.73 (0)	0.22 ± 0.51 (0)
2	28	0.14 ± 0.45 (0)	0.96 ± 0.84 (1)	0.07 ± 0.26 (0)	0.11 ± 0.31 (0)	1.75 ± 0.84 (2)	0 ± 0 (0)	1.89 ± 0.64 (2)
3	14	0.08 ± 0.28 (0)	0.38 ± 0.65 (0)	0.58 ± 0.9 (0)	1.77 ± 0.6 (2)	2.15 ± 0.8 (2)	1.08 ± 1.04 (1)	2.69 ± 0.63 (3)

Data is presented as number of days (percent of days) and mean ± SD (median).

##### Biochemical analysis for safety

**Figure 4:**
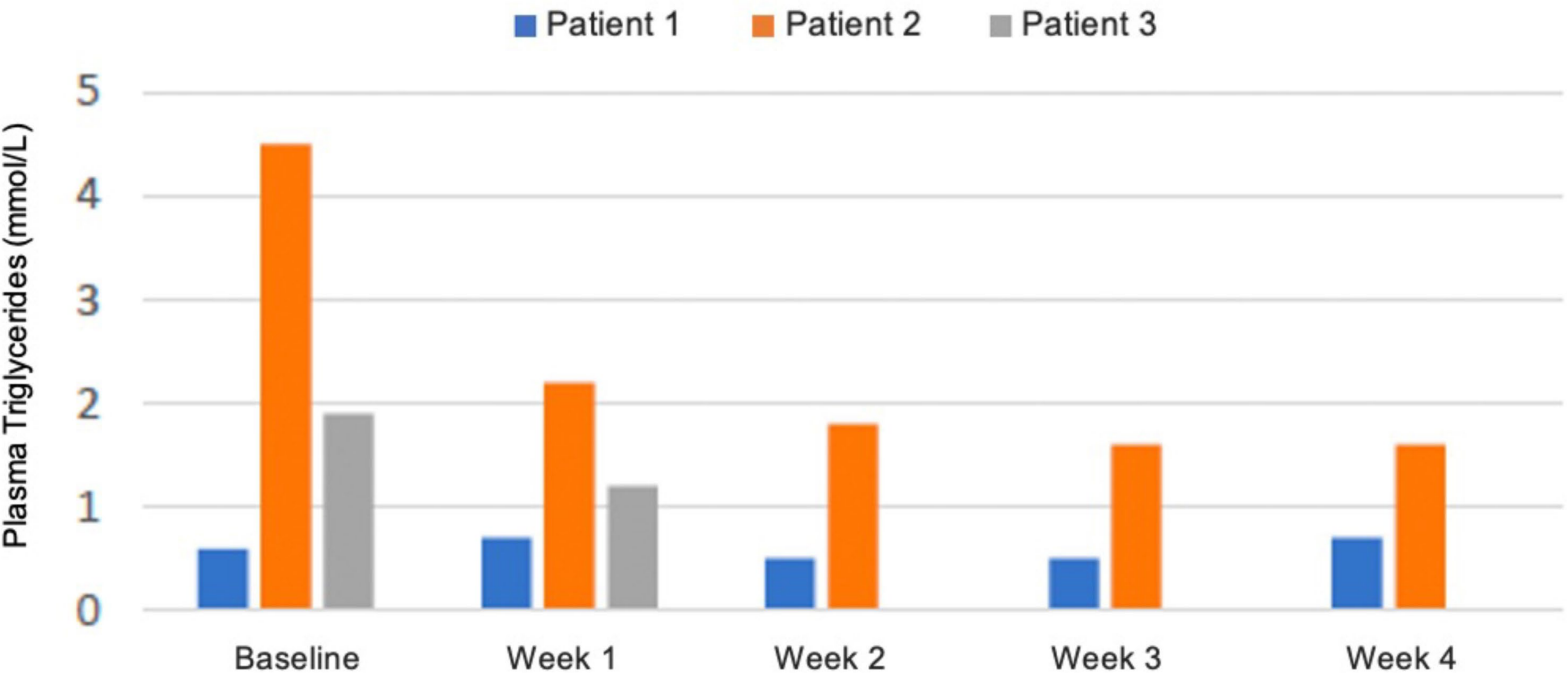
Plasma levels of triglycerides in mmol/L from baseline to week 4. Patient 3 dropped out of the study before blood measurements were taken after 2 weeks. Blood samples were taken postprandial and not in a fasted state. *Reference values for adults: triglycerides <2.0 mmol/L*.

**Figure 5:**
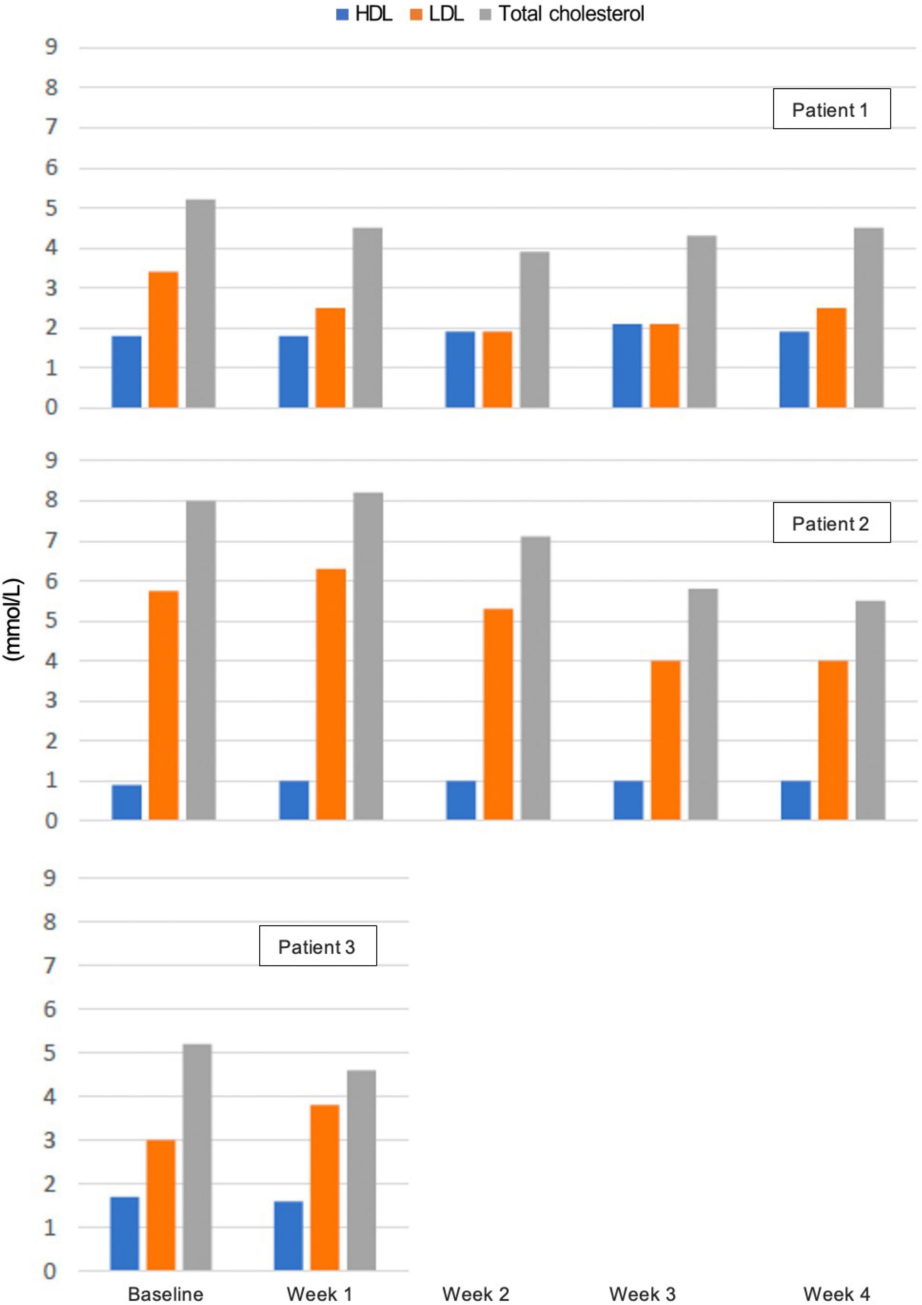
Plasma levels of total cholesterol, high-density lipoprotein (HDL), and low-density lipoprotein (LDL) in mmol/L from baseline to week 4. Patient 3 dropped out of the study before blood measurements were taken after 2 weeks. Blood samples were taken postprandial and not in a fasted state. *Reference values for adults: total cholesterol <5.0 mmol/L, HDL > 1.2 mmol/L (women) / >1.0 mmol/L (men), LDL < 2.6 mmol/L*.

**Figure 6:**
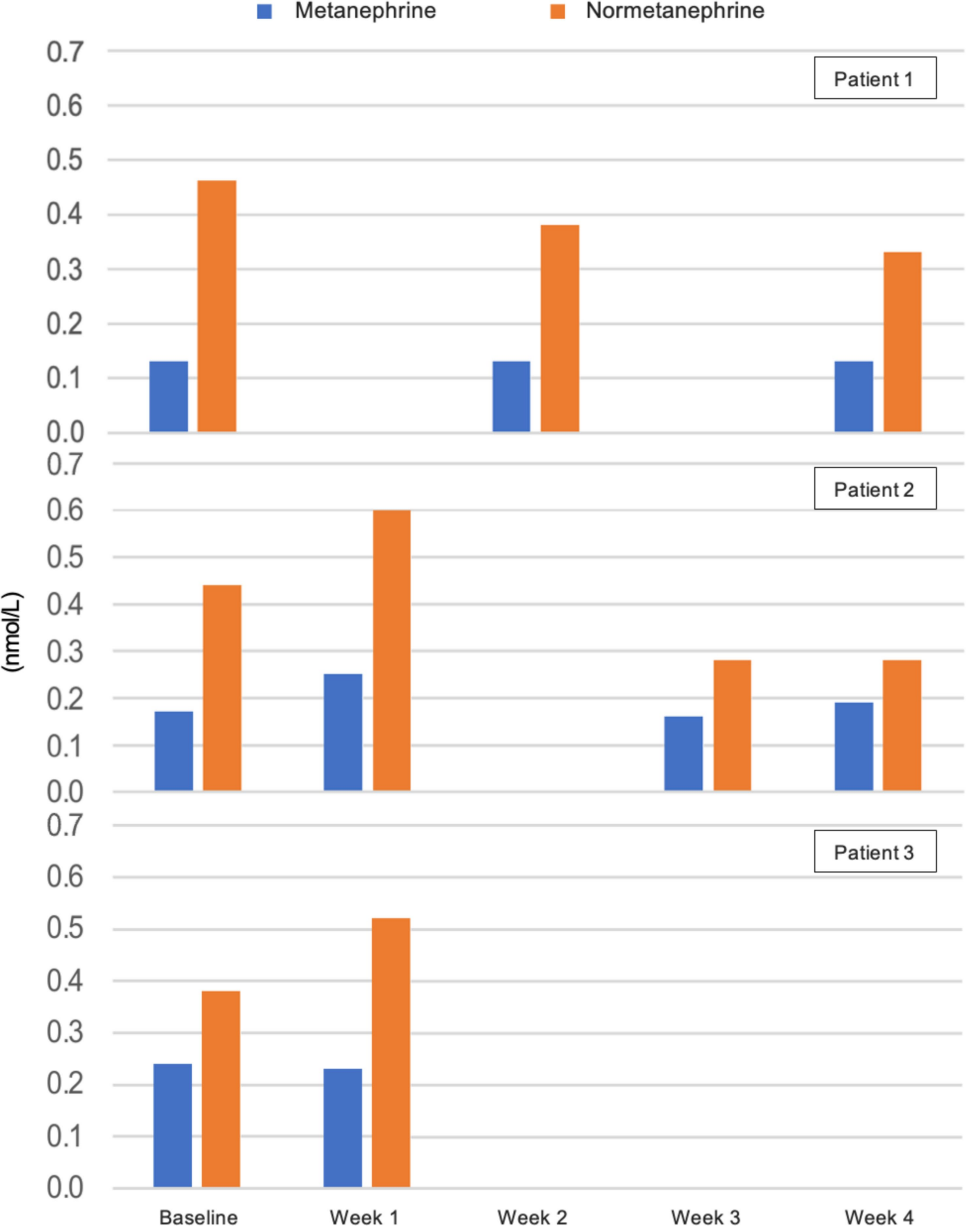
Plasma levels of metanephrine and normetanephrine in nmol/L from baseline to week 4. Results lacking at weeks 1 and 3 in patient 1 and in week 2 in patient 2 are due to haemolysis of blood samples. Patient 3 dropped out of the study before blood measurements were taken after 2 weeks. *Reference values for adults: metanephrine <0.34 nmol/L, normetanephrine <0.76 nmol/L*.

**Figure 7:**
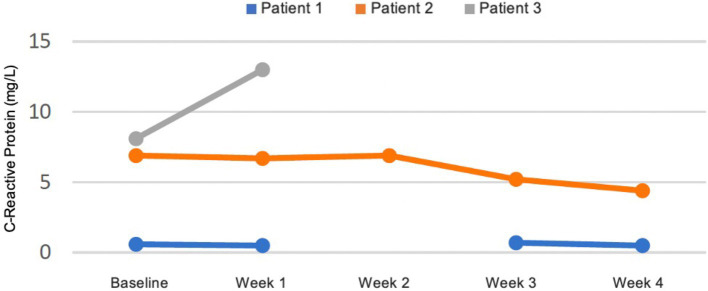
Plasma levels of C-reactive protein (CRP) in mg/L from baseline to week 4. Results lacking at week 2 in patient 2 are due to haemolysis of blood samples. Patient 3 dropped out of the study before blood measurements were taken after 2 weeks.

**Figure 8:**
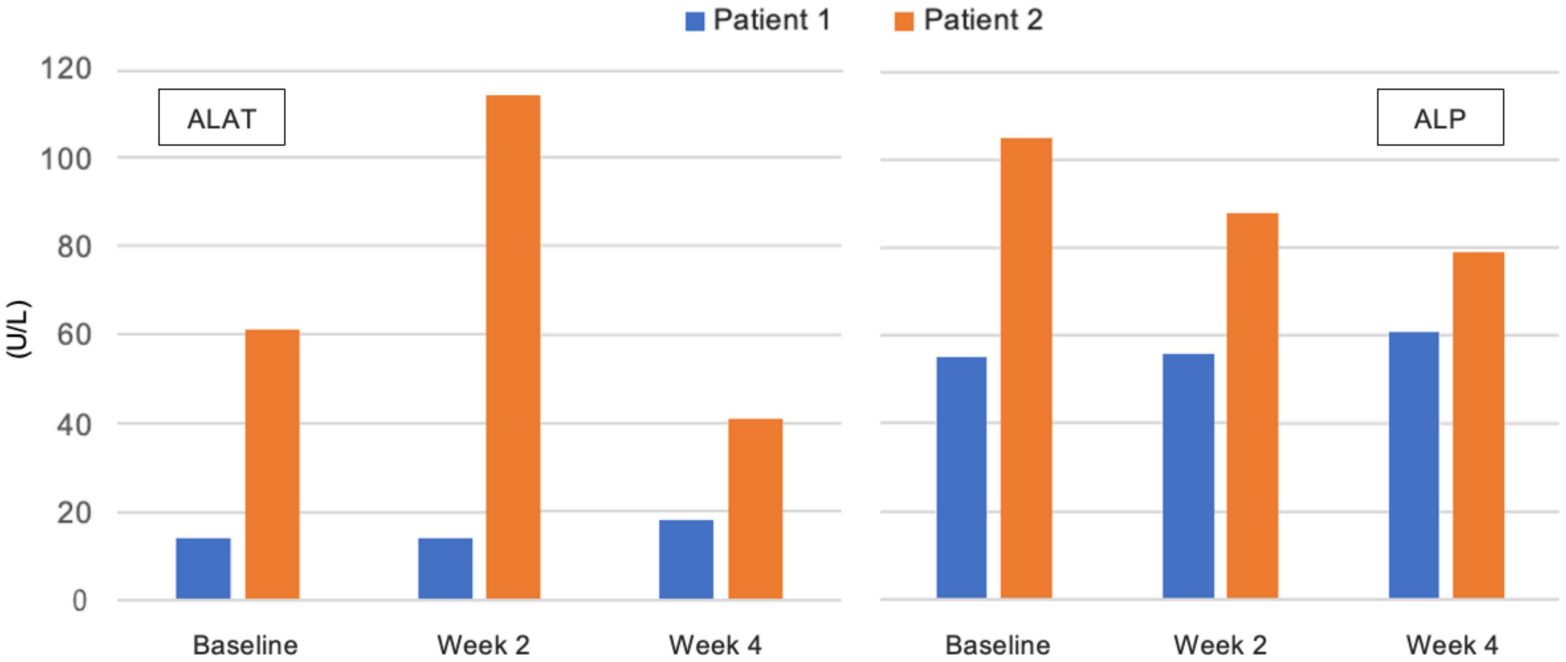
Plasma levels of alanine aminotransferase (ALAT) and alkaline phosphate (ALP) in U/L from baseline to week 4. Patient 3 dropped out of the study before blood measurements were taken after 2 weeks. *Reference values for adults: ALAT 10-45 U/L, ALP 35-105 U/L*.

There were no significant changes in sodium, potassium, magnesium, creatinine, phosphate, and bilirubin from baseline and measurements were within reference values during the intervention. Plasma triglycerides were elevated in patient 2 at baseline (4.6 mmol/L), normalised within the reference value of <2.0 mmol/L after 2 weeks, and was 1.6 mmol/L at the end of week 4 (Figure 4). In the same patient, total cholesterol decreased from 8.0 mmol/L at baseline to 5.5 mmol/L after 4 weeks and LDL decreased from 5.7 to 4.0 mmol/L. In patient 1 total cholesterol decreased from 5.2 to 4.5 mmol/L and LDL from 3.4 to 2.5 mmol/L, while HDL increased slightly from 0.9 to 1.0 mmol/L. In patient 3 total cholesterol decreased from 5.2 to 4.6 mmol/L after 1 week, but LDL increased from 3.0 to 3.8 mmol/L (Figure 5). Due to patient 3 dropping out of the study after 2 weeks, only blood measurements after week 1 were taken, as the patient did not come in for measurements at the end of week 2. Plasma levels of metanephrine and normetanephrine were within reference values in all three patients (Figure 6). CRP was normal in patient 1, elevated in patients 2 (7 mg/L) and 3 (8 mg/L), and increased from baseline to week 1 (13 mg/L) in patient 3 and decreased from baseline to week 4 (4 mg/L) in patient 2 (Figure 7). ALAT was elevated in patient 2 at baseline (62 U/L) and almost doubled (115 U/L) at week 2 but normalised at week 4 (41 U/L). ALP was within reference values in both patients 1 and 2, and decreased from 105 to 79 U/L in patient 2 (Figure 8).

#### Acceptance of treatment

All patients were compliant with the intervention and the treatment was therefore accepted in 100% of intervention days by all patients, even though patient 3 dropped out of the study after 2 weeks. The dietary intervention and daily measurements/registrations were found not demanding by patient 1, and slightly demanding by patient 2. Patient 3 found the dietary intervention moderately demanding in 64% of days (nine of 14), quite to very demanding in the remainder of days (36%, five of 14), and the daily measurements/registrations quite to very demanding in 21% of days (three of 14). Patients 1 and 2 completed the nutrition intake registration on 100% of the days and patient 3 on 93% of the days (13 of 14). The mean daily nutrition intake during the intervention for all patients can be seen in Table 4.

**Table 4:** Mean daily nutrition intake for all patients during the intervention.

Patient	Energy (kcal)	Fat (g)	Carbohydrate (g)	Sugars (g)	Dietary Fibre (g)	Protein (g)	Fat E%	Carbohydrate E%	Protein E%
1	1,937	152.5	26.5	14.8	27.7	94.6	69.2	8.4	20.1
2	1,628	126.0	13.2	8.6	21.3	99.2	67.2	6.1	25.3
3	1,582	117.3	15.6	10.2	12.6	91.0	65.9	5.7	24.0
**Mean**	**1,716**	**131.9**	**18.4**	**11.2**	**20.5**	**94.9**	**67.4**	**6.7**	**23.1**

E%: energy percent.

### Exploratory outcomes

**Figure 9:**
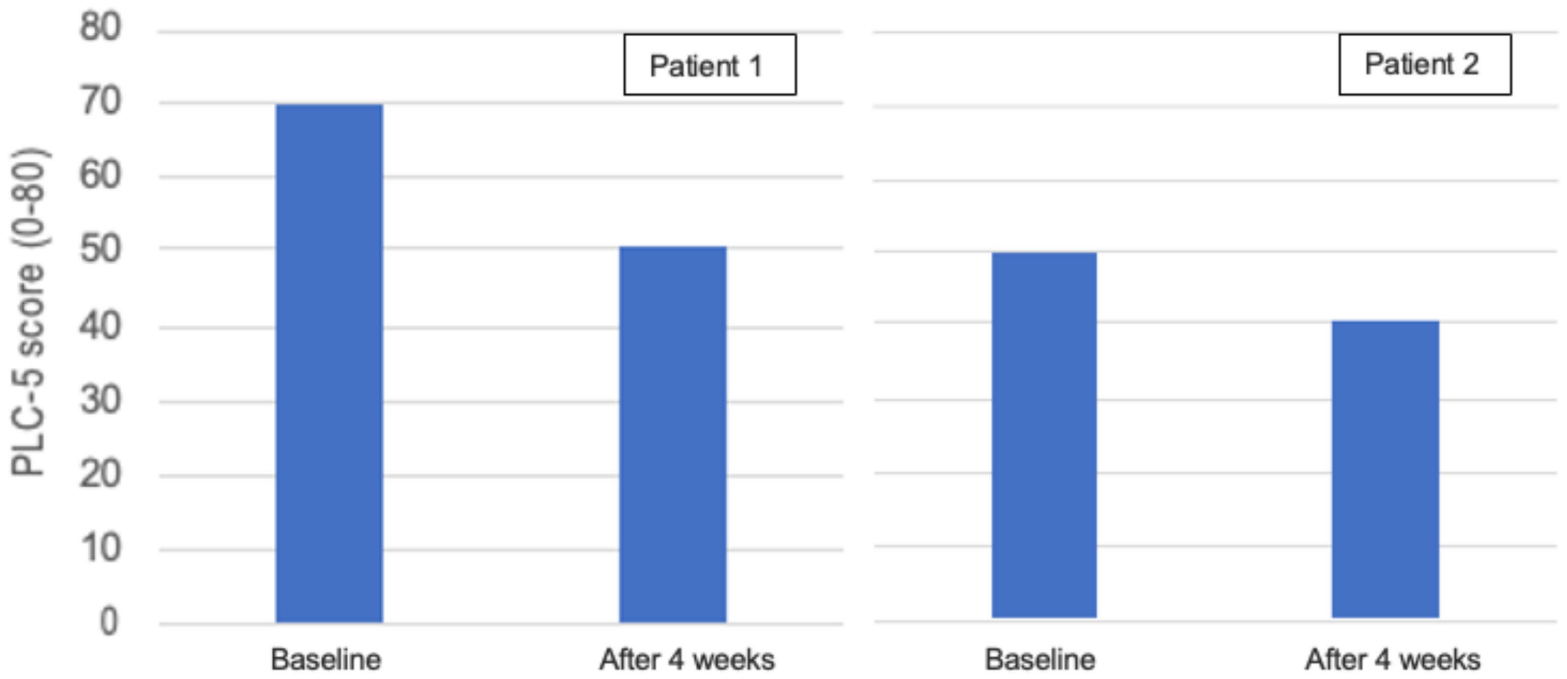
PTSD Checklist for DSM-5 (PCL-5) in patients 1 and 2 at baseline and after 4 weeks. PCL-5 is a 5-point scale with 20 questions (119). Each question is answered based on the frequency of experiencing a particular symptom, ranging from 0 (not at all) to 4 (very often). The minimum value is 0 and the maximum value is 80, the lower the score the better.

**Figure 10:**
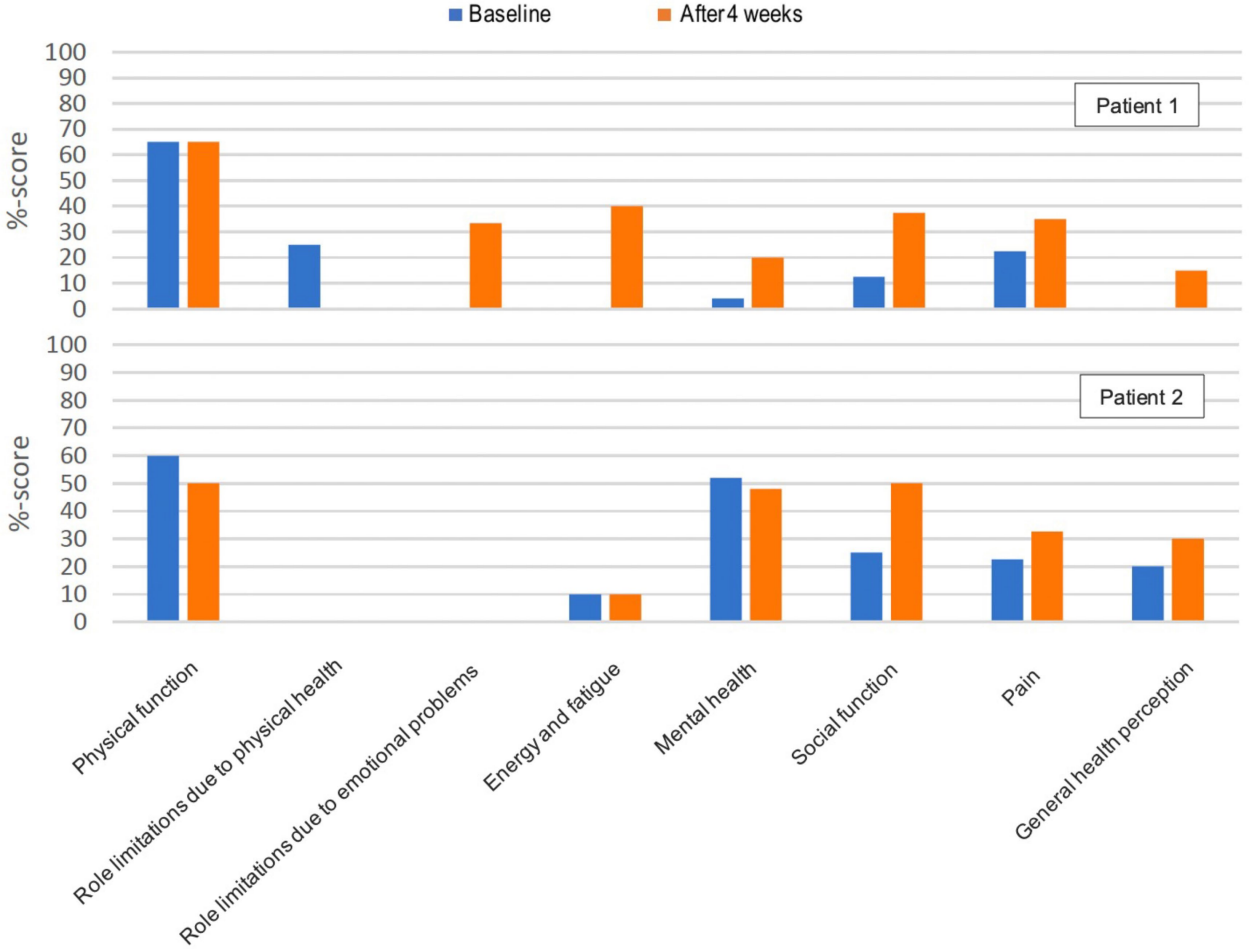
Health-related quality of life (RAND-36) (120) in patients 1 and 2 at baseline and after 4 weeks. The score represents a percentage of the total possible score, ranging from 0 to 100%. Higher scores are better, and 0 is worse.

Only patients 1 and 2 completed the PCL-5 and RAND-36 after the intervention, due to patient 3 dropping out of the study, and the investigator could not get the patient to complete the last assessments. In patient 1 the PTSD symptoms measured with PLC-5 went from a score of 70 to 50 and in patient 2 from a score of 50 to 40 in 4 weeks (Figure 9). Both patients reported an improved overall quality of life during conversations with the investigator. In patient 1 six of eight of the RAND-36 subscales improved (Figure 10). Role limitations due to emotional problems, energy and fatigue, and general health perception increased from 0 to 33%, 40 and 15%, respectively. The patient described significantly less pain due to fibromyalgia and on the RAND-36 subscale pain, there was an improvement of 13%. Social function increased by 25% and mental health by 16%. Physical function did not change and role limitations due to physical health decreased by 24%. Three of eight of the RAND-36 subscales improved in patient 2: social function by 26%, pain by 10%, and general health perception by 10% (Figure 10). Role limitations due to physical health, role limitations due to emotional problems, and energy and fatigue did not change, physical function decreased by 10% and mental health by 3%.

#### Weight

All patients lost weight during the intervention. Patient 1, who had a BMI in the healthy weight range at baseline (22.6), lost 0.2 kg, while patients 2 and 3, who were obese at baseline (BMI 32.0 and 32.2), lost 5.1 kg in 4 weeks and 3.5 kg in 2 weeks, respectively. Changes in weight and BMI from baseline until the end of the intervention are seen in Table 5.

**Table 5:** Weight and body mass index (BMI) changes from baseline until the end of the intervention in all patients.

Patient	Weight Baseline (kg)	BMI Baseline	Weight Week 1 (kg)	Weight Week 2 (kg)	Weight Week 3 (kg)	Weight Week 4 (kg)	BMI Week 4	Weight Difference (kg)	BMI Difference
1	63.1	22.6	63.1	62.9	63.6	62.9	22.6	−0.2	0
2	85.0	32.0	81.6	80.6	79.9	79.9	30.1	−5.1	−1.9
3	92.0	32.2	88.6	88.5	−	−	−	−3.5	−0.8

## Discussion

### Feasibility outcomes

#### Recruitment of patients

Being the first study investigating KD-KS in PTSD patients, the results can only be compared with PTSD studies examining other interventions. For the recruitment feasibility criteria to be fulfilled, we set a goal of ≥60% eligible patients to be included, based on previous PTSD pilot studies (114, 115) using a lower limit of the 95% confidence interval of ≥60% included eligible patients. The study by Oehen et al., looking at MDMA-assisted psychotherapy in chronic PTSD, included 74% [95% CI: 51 to 88%] of eligible patients (115), while the study by Hall et al., examining community-based exercise in older veterans with PTSD, included 20% [95% CI: 16 to 26%] of eligible patients (114). In our study three of four eligible patients were included (75% [95% CI: 30 to 95%]). We therefore surpassed our goal of including ≥60% eligible patients by 15% but did not reach the aim of including 10 patients in total.

In this study, the treating physician performed pre-screening of their patients and referred eligible patients to the investigator for a full screening visit. This recruitment strategy was approved by REK and was intended to inform the patients about the study and inquire about interest, without patients feeling pressured to participate. The ethics approval stated that the investigator could not contact patients directly before a referral was made. We therefore depended on clinicians pre-screening patients for the study. Southern Oslo DPC at Oslo University Hospital receives patients from three Southern districts in Oslo, Norway. It was estimated before the study that a large proportion of the patients would be eligible, yet only four patients were referred by their treating physician to the investigator. The aim was to continue including patients until 10 patients completed the full 4-week intervention, but due to lack of referrals, this was not achievable within the timeframe of the study. It is unlikely that insufficient recruitment for the study is due to a small patient population to recruit from. We speculate that the main reason for the lack of referrals is that patients did not become aware of the ongoing study and were not asked if they were interested. Even though the investigator reminded clinicians about the study, held presentations and handed out material about the study, busyness likely made them forget to pre-screen their patients, and it is also possible that some found it inappropriate to inform patients about the study during treatment sessions. A large number of clinicians in this clinic are psychologists, and they may be less knowledgeable and perhaps more sceptical about unproven biological treatments. The investigator was not a full-time employee at DCP and was only present at the clinic when seeing patients and attending meetings, conferences and presentations, which could have contributed to the study being easier to forget by the clinicians, than if a full-time colleague was running the study. There could also be other reasons that the clinicians did not want their patients to participate. Patients could also not wish to participate due to diet preferences, the extent of the diet intervention, and measurements/registrations that should fit into everyday life, often with other family members, concurrent with debilitating PTSD. The clinical experience at DPC is that many PTSD patients use food for comfort and emotional regulation. The hospital also has many patients with refugee backgrounds, some who have experienced torture, or other patients who have experienced events that make repeated finger-pricker blood tests a reason not to participate. Five PTSD patients not in treatment at DPC contacted the investigator with interest in participating in the study. Unfortunately, these patients could not be enrolled in the study due to the ethics-approved protocol where only outpatients in treatment at DPC could be included. The study team did also receive requests from PTSD patients and clinicians both in Europe and in the USA, asking for participation in the study. It is therefore our impression that there is a large interest and possible need for this intervention in the PTSD population. A limitation of this study is that clinicians did not register data from the pre-screening and therefore the actual number of eligible PTSD patients at DPC during the inclusion period is not known.

All participants in this study were women, who have a 2 to 3 times greater chance of developing PTSD than men after traumatic events, with an approximate 2:1 ratio of women to men with diagnosed PTSD (122, 123). Women also seem to seek treatment more often than men (124). An overrepresentation of women in the study was expected. The mean age was 49.3 ± 4.0 years and the mean PCL-5 score for severity of PTSD symptoms was 59 ± 10. In a randomised controlled trial (RCT) with female veterans with PTSD (*n* = 104), who experienced sexual trauma during service, the mean age was 48.4 ± 11.1 years. The study included two different treatment groups with baseline mean PCL-5 scores: 50.8 ± 11.7 (*n* = 56) and 50.4 ± 12.1 (*n* = 46) (125). Taking into account the small sample size, the population in this study is similar in age and severity of PTSD symptoms to larger populations with female PTSD patients.

#### Maintaining ketosis

Patients 1 and 2 attained ketosis on day 2, and patient 3 on day 1. At least one b-BHB measurement was ≥0.5 mmol/L in all patients on the first day of the intervention, on the first (morning), second (mid-day), or third (evening) measurement of the day. For maintaining ketosis criteria, we set a goal of ≥75% of days in ketosis since ketosis was attained, based on a study by McDonald et al. in epilepsy patients (113). Ketosis was maintained in 87% of the intervention, meaning that this goal was achieved and surpassed by 12%.

Patient 3 maintained ketosis in 100% of days and patient 1 had one day not yet in ketosis on intervention day 1 (mean daily b-BHB 0.43 mmol/L). Patient 2 maintained ketosis in 71% of days (20 of 28 days) despite being 100% compliant with KD-KS, according to daily nutrition registration and conversations several times a week with the investigator. This patient had the highest BG and lowest b-BHB levels (mean b-BHB 0.6 mmol/L, highest b-BHB 1.1 mmol/L) (Figure 2) out of the three patients. Especially in the last week of intervention, b-BHB levels started to drop while BG levels stayed the same, and this seemed to be caused by adding Sukrin^®^ Fibre Bread (low-carb bread, per 100 g: 163 kcal, 8.4 g fat, 2.5 g carbohydrates, 21 g fibre, 8.7 g protein) to the diet. Baseline triglycerides, total cholesterol, and LDL were elevated in this patient, baseline BMI was 32 and the patient had prediabetes, metabolic syndrome, and hypothyroidism, which explains the elevated blood lipids at baseline. b-BHB levels were lower in this patient after a long overnight fast or on days with reported lower food intake, and on days with higher fat intake b-BHB levels were higher. The investigator encouraged the patient to consume more of the provided food products with a high-fat content, but unfortunately, the patient did not find them palatable. Patient 1 achieved the highest b-BHB levels (mean daily b-BHB 2.2 mmol/L) and had the lowest BG throughout the study (Figure 2). This patient was normal weight (baseline BMI 22.6), baseline triglycerides were within the reference range and baseline total cholesterol and LDL were slightly above reference values. This patient had the highest fat intake (Table 4) and primarily consumed the food products provided in the study. b-BHB levels increased in this patient on days with low food intake. In patient 3 mean daily b-BHB was 1.1 mmol/L with the highest measurement of 2.1 mmol/L. During week 1 mean daily b-BHB was on average approximately 1.0 mmol/L and after increasing fat intake b-BHB levels increased to >2 mmol/L but decreased again to approximately 1.0 mmol/L when the patient could not sustain the higher fat intake. This patient had a baseline BMI of 32.2, normal triglycerides, and total cholesterol and LDL just above the reference range.

In this study, patients were encouraged to predominantly consume the ketogenic ready meals and food products provided, but also had the option to prepare their own ketogenic meals and snacks with guidance from the investigator. Patient 1 primarily consumed the food products provided in the study, while the other patients consumed few of the provided food products and primarily prepared their own ketogenic meals. All patients consumed their exogenous ketone supplement Ketostart^®^ throughout the day. Despite the freedom to choose preferred food items and compose their own meals, ketosis was maintained in 87% of the intervention, which is a strength of this study. With many PTSD patients using food for comfort and emotional regulation, it was a success that ketosis could be maintained to this extent during a 4-week intervention in two patients, and 2 weeks in one patient.

Another strength is that b-BHB and BG were measured three times daily with GKI-Bluetooth Blood Glucose & Ketone Meter^®^ (Keto ***-*** Mojo Europe B.V., Amsterdam, Netherlands), and only 2% of measurements were missed. b-BHB and BG measurements were uploaded to an app on the patient’s phone, which synchronised with MyMojoHealth’s online platform for practitioners. The investigator and study team could follow the measurements in real-time, which allowed reaching out to patients if measurements were missed, or measurements showed a lack of compliance. Furthermore, GKI-Bluetooth Blood Glucose & Ketone Meter^®^ meets the accuracy criteria in ISO 15197: 2013 standard (126, 127) and is commonly used in other trials (65, 128–131). ISO 15197: 2013 does not specify the accuracy of b-BHB measurements. It should be noted that the accuracy of BG measurements may be affected by biochemical changes that often occur in ill patients and by the medication they receive. Little is known about the influence of these factors on b-BHB measurements (132).

#### Occurrence of serious adverse reactions and adverse reactions to the ketogenic diet supplemented with *β*-hydroxybutyrate salt

For the occurrence of SARs and ARs to the KD-KS we set a goal of ≤5% of intervention days with SARs in 100% of patients and ≤ 30% of intervention days with ARs in ≥75% of patients, based on the results from two Cochrane reviews by Martin-McGill et al. in epilepsy patients (116, 117). There were no SARs and a total of 70% of intervention days with ARs, and therefore the occurrence of ARs to the KD-KS were not within the predefined goals.

Headache followed by fatigue were the most frequent and the strongest perceived ARs by the patients. When entering the study two patients suffered from fatigue, all three patients from fibromyalgia, two from headache, and one from bipolar disorder type 2. Headache, particularly migraine, and fatigue are frequent comorbid symptoms of PTSD, fibromyalgia, and bipolar disorder 2 (133–135). In patient 1 there was a 40% improvement of the RAND-36 subscale: energy and fatigue, and in patient 2 there was no change. Pain improved by 13% in patient 1 and by 10% in patient 2. Headache and fatigue are transient symptoms, often referred to as “keto flu”, that can occur when adapting to a KD and are most likely due to the sudden decrease in carbohydrates and sugars, and the initial increased urinary excretion of electrolytes, especially sodium, and potassium (136, 137). It is not possible to distinguish between headache and fatigue due to PTSD and comorbidities, and ARs caused by the intervention. A significant improvement from baseline in energy and fatigue in patient 1, no change in patient 2, and improvement in pain in both patients, suggests that the frequent occurrence of headache and fatigue during the intervention are mainly due to pre-existing conditions. Percent of intervention days with other ARs were constipation 39%, stomachache 33%, nausea 29%, dizziness 29%, and diarrhoea at 7%, and the participant-perceived degree of these ARs was low, with the exception of stomachache in patient 3 which was perceived as moderate. This is consistent with the results of two Cochrane reviews by Martin-McGill and al., examining the KD for drug-resistant epilepsy, where the most commonly reported ARs to the KD were gastrointestinal symptoms (116, 117).

There was a beneficial effect on plasma triglycerides, total cholesterol, and LDL, especially in patient 2, where significantly elevated levels normalised. LDL increased from 3.0 to 3.8 mmol/L in patient 3, but due to the patient dropping out of the study after 2 weeks, there was only one measurement 1 week after baseline, and it is unknown if this value would decrease again, if the patient had completed the 4-week intervention. The KD can result in an often-temporary increase in LDL, but long-term effects are often an increase in HDL and a decrease in LDL (138, 139). An exception is a subgroup of lean individuals called “Lean Mass Hyper-Responders”, that responds to carbohydrate restriction with a larger increase in LDL (140). It should be considered that all blood samples were taken postprandial and not in a fasted state, which affects the levels of circulating blood lipids. It would have been optimal to measure fasting levels of blood lipids to eliminate the effect of the previous high-fat meal, but measurements were taken in connection with appointments at DPC at any time of day, and it was not feasible to ask the patients to fast until later in the day.

CRP at baseline in patients 2 (7 mg/L) and 3 (8 mg/L) was indicative of low-grade inflammation seen in obese patients with metabolic syndrome (141). CRP was ≤1 mg/L in patient 1 with a healthy BMI. There was an increase in CRP in patient 3 from baseline (8 mg/L) to week 1 (13 mg/L) which could indicate an incipient infection. In patient 2 CRP decreased from 7 to 4 mg/L in 4 weeks, which may be due to the anti-inflammatory effects of the KD-KS (22, 94, 95). For unknown reasons, ALAT increased in patient 2 from 62 to 115 U/L after 2 weeks and decreased to 41 U/L, within reference range, after 4 weeks. In the same patient and period, ALP decreased from 105 to 79 U/L.

#### Acceptance of treatment

For the acceptance of treatment criteria, we set a goal of ≥75% of patients accepting the treatment in ≥75% of intervention days (113). All patients accepted the treatment in 100% of days, even though patient 3 only completed a 2-week intervention, hence this goal was met. With this small sample size of three patients, the dropout rate was 33%. Due to the lack of diet intervention studies in this population, more research is needed to establish the dropout rates in larger powered studies. A high dropout rate was expected despite close monitoring. PTSD is considered the most severe and pervasive trauma disorder, and patients experience numerous distressing symptoms and limitations that significantly impact their lives (6, 10, 142). A strength of the study is that 2 of 3 (67%) patients found the dietary intervention and daily measurements/registrations not demanding to slightly demanding, even though baseline severity of PTSD symptoms (PCL-score) were 70 and 50, respectively. The patient who dropped out of the study found the dietary intervention moderately demanding in 64% of days, quite to very demanding in 36% of days, and the daily measurements/registrations quite to very demanding in 21% of days. The reason given by this patient for withdrawing from the study, was because all the procedures, planning, and cooking became too demanding. The patient did not like any of the food products provided, adding to the burden of planning meals, cooking, measuring food/fluid, and registering daily nutrition intake. Before entering the study, the patient struggled with fatigue, and some days could be particularly challenging, making ready meals and products preferable. After the first intervention week, the patient stated that she found it exhausting but wanted to continue in case the first week was the toughest. She felt increasingly fatigued in week 2 and ultimately chose to withdraw after day 14.

Due to import rules between the United Kingdom and Norway and no other known provider of ketogenic ready meals shipping to Norway from elsewhere, the variety of ready meals offered in this study was limited to six meals that were all vegan. Two were soups, two contained jackfruit and two contained mushrooms. The ready-to-drink ketogenic formulation K.Flo^®^ (vanilla flavour) and semi-solid ketogenic formulation K.Yo^®^ (vanilla/chocolate flavour), were intended for breakfast, snack/dessert, and/or meal replacement. It was intended to provide the patients with complete nutrition to ease the intervention and help with compliance. To prevent dropouts in future studies in this population, a larger variety of ketogenic ready meals must be provided to suit different preferences and ease the burden of food shopping and cooking. In this study, all nutrition and fluids were registered daily. When all meals are provided this is less of a burden for the patients, but when patients prepare ketogenic meals themselves, daily nutrition registration can become too strenuous. For future studies, choosing the most important data to collect and lessening registrations by the patient could potentially lead to more patients completing the intervention.

Continuous glucose monitors have now been on the commercial market for some time and continuous ketone monitors and consumer biowearables that can measure glucose, ketones and other substrates like lactate and ethanol are on their way to market. Especially in the subpopulation of PTSD patients who have experienced bodily harm and torture, finger-pricking to obtain daily ketone and glucose measurements can be triggering and not feasible. To ease the burden of measurements and to be able to include patients who do not want to perform finger-prick blood measurements, a continuous dual ketone/glucose monitor would be optimal for future trials. This will also provide the researchers with more data and may ease compliance in real-time when both patients and investigators can follow the daily glucose and ketone fluctuations. For patients not willing or able to follow a KD intervention, using exogenous ketone supplementation with other more moderate diet protocols or the patients’ habitual diet, is also something that should be examined in future trials.

### Exploratory outcomes

The PTSD symptoms measured with PLC-5 decreased by 20 points (70 to 50) in patient 1 and by 10 points (50 to 40) in patient 2. A change of 5–10 points is considered a reliable change (more than random variation) and a change of minus 10–20 points indicates a clinically meaningful change (143). Patient 1 was concurrently in psychological therapy, so any potential isolated treatment effect of the KD-KS intervention cannot be estimated. The patient reported various influences on PTSD symptoms that were perceived positively. She described improved sleep with significantly fewer nightmares, increased energy (Figure 10), and a better ability to concentrate in conversations without “spacing out.” Her nightmares returned on days when b-BHB levels were lower (approximately 1.0 mmol/L), due to the introduction of Sukrin^®^ Fibre Bread into the diet. She also experienced more pain on those days, but she thought that stomach discomfort from the high fibre content in the bread made her more sensitive to other bodily pains. Patient 2’s psychologist was on sick leave throughout the study, so it can be argued that there may be a more direct connection between ketosis and the reduction in PTSD symptom severity. The patient consistently had lower levels of b-BHB than patient 1 throughout the study. Patient 2 described that her nightmares were less intense than they used to be. She expressed a sense of increased clarity and energy in her mind, and, overall, she felt better. Hypothetically, diet-induced ketosis could be a complementary therapy that potentially can enhance the benefits of psychological therapy by helping the patient stay within the window of tolerance, neither hyperaroused nor hypoaroused, where they are receptive to psychological therapy and able to process trauma (88, 89, 98–100), but this hypothesis needs to be tested in future studies measuring an effect. Reasons for patient 1 experiencing a greater improvement in PTSD symptoms may include a higher initial score, making the change easier to detect. This patient was concurrently undergoing psychological therapy and consistently achieved higher b-BHB levels throughout the study. There is no established knowledge about the optimal level of ketosis for the best therapeutic effect in various neurological and mental disorders, but some studies show a dose–response relationship (65) and there might be an individual threshold to surpass. There is a direct relationship between the concentration of ketones in the blood and the proportion of ketones oxidized to ATP (144). Higher levels of ketones supply the brain with more energy, as an alternative fuel source to glucose, and in addition to being an energetic metabolite, BHB is also a signalling metabolite that affects epigenetic gene regulation and cellular function and has important neuroprotective effects (39, 40).

One can speculate that quality of life measured with RAND-36 also may be correlated with increasing levels of b-BHB. This correlation may be indirect, as the reduction of PTSD symptoms naturally influences it. However, it may also be direct by increasing energy and reducing pain and inflammation (94, 95, 145, 146). During conversations with the investigator, both patients reported improved overall quality of life. At baseline, patient 1 had very low scores on seven of eight of the RAND-36 subscales, where three subscales: role limitations due to emotional problems, energy and fatigue, and general health perception, was 0% which represents the poorest possible function. These three subscales increased to 33, 40, and 15%, respectively and one can argue that an increase in quality of life from 0 to 30% may be more noticeable and valuable for the patient, than a change from 30 to 60%. During this relatively short intervention period of 4 weeks, the six subscales related mostly to mental factors: mental health (+16%), role limitations due to emotional problems (+33%), social function (+25%), general health perception (+15%), energy and fatigue (+40%), and pain (+13%), improved in patient 1, while the two subscales related to physical factors: physical function (+0%) and role limitations due to physical health (-24%), stayed constant or decreased. This suggests that the potential beneficial effects of ketosis that occur rather fast, are predominantly mental, while beneficial effects on physical factors, especially role limitations due to physical health, are not seen during this 4 weeks intervention. This is consistent with trials in weight loss, and in neurological and mental disorders, where ketosis is shown to be beneficial on mental factors (66, 83, 147–149), while studies also show that physical factors can decrease in the initial weeks of “keto-adaptation” and beneficial effects on physical factors, e.g., physical function and performance, happen first after 3–4 weeks and can continue to improve up to 6 months and potentially longer (44, 145, 150–153).

In patient 2 several of the baseline scores were higher than in patient 1, indicating better functions in the subscales: mental health (+44%), social function (+12%), general health perception (+20%), and energy and fatigue (+10%) in this patient, but the baseline score for role limitations due to physical health was lower (-24%). Three of eight subscales improved during the intervention: social function (+26%), pain (+10%), and general health perception (+10%), while role limitations due to physical health and role limitations due to emotional problems remained on a score of 0%, energy and fatigue remained on a score of 10%, physical function decreased by 10% and mental health by 3%. During the last assessment in the study, the patient mentioned that the last week of intervention had been a bit mentally challenging, and motivation had been lower without knowing exactly why. She suggested that it may have influenced the responses to the psychological measurements at the end of the study. This is most noticeable on the subscale: energy and fatigue, where she noted feeling more energised throughout the intervention.

Even though the baseline score for social function was 12% higher in patient 2, the increase in this subscale was 26 and 25% in patient 2 and patient 1, respectively. There was also a similar improvement in both patients in general health perception by 20 and 15%, and in pain by 13 and 10% in patient 2 and patient 1, respectively. Energy and fatigue did not improve in patient 2 while it improved by 40% in patient 1. The difference in the baseline score by +10% in patient 2 could have made a slight difference and patient 2 stated being more energised for the first 3 weeks of intervention. Role limitations due to physical health did not change from 0% in patient 2, while it decreased from 24 to 0% in patient 1. Physical function did not change in patient 1 but decreased by 10% in patient 2. Role limitations due to emotional problems remained at 0% in patient 2 and increased by 33% in patient 2. In patient 2 mental health decreased by 3% while improving by 16% in patient 1. This all shows some similarities in the improvement on the RAND-36 in both patients, but also differences that could depend on various factors such as different baseline function on RAND-36, therapy versus no therapy, differences in metabolic health and comorbidities, and the severity of PTSD symptoms at baseline, which was higher in patient 1 who also had a two times reduction in PTSD symptoms compared to patient 2, and also in differences in level of ketosis.

Like patient 1, patient 2 experienced an increase in symptoms in the days following the introduction of Sukrin^®^ Fibre Bread as a variation to seed crackers (Norwegian crispbread). On the days when the bread was consumed, the patient fell out of ketosis. Throughout the study, patient 2 had lower levels of b-BHB than patient 1, but there may have been a beneficial effect even at low to moderate b-BHB levels (0.5–1.0 mmol/L). When entering the study, patient 2 suffered from chronic joint pain in the fingers and daily intense headaches. After the first week on KD-KS, joint pain and swelling significantly decreased, and the intensity of headaches was reduced, with some days being completely symptom-free. The KD is investigated as an intervention in migraine and studies are showing promising results (101, 102). The known anti-inflammatory properties of the KD can be why this patient experienced less joint pain and swelling during the intervention (94, 95).

## Conclusion

To the best of our knowledge, this feasibility study is the first clinical human trial examining a KD supplemented with exogenous ketones as a treatment intervention in patients with PTSD. Over 4 weeks, we observed that KD-KS led to clinically meaningful improvements in PTSD symptoms and health-related quality of life. In total, three of four predefined feasibility criteria were achieved, but the aim of including 10 patients was not reached. There is a large uncertainty to whether there were eligible patients not referred to the study. Ketosis was achieved within one to two days and maintained during the intervention, there were no SARs, the occurrence of ARs was high and not within the predefined goals, but the participant-perceived degree of ARs was low. A large proportion of registered ARs were likely due to pre-existent comorbidities and symptoms of PTSD. Despite this, the treatment was accepted by all patients in 100% of intervention days even though one patient dropped out of the study after 2 weeks due to exhaustion and fatigue.

### Recommendations for future research

Even though the sample size is small, the knowledge obtained in this study is important for the planning of future studies with KD and exogenous ketone interventions in this patient group. Further feasibility and pilot studies should be performed with larger sample sizes to determine feasibility and safety before planning future RCTs. Due to the complexity of the patient group with many comorbidities and confounders affecting the results, future RCTs must be carefully planned, and the sample sizes must be powered to examine a possible effect of ketosis on PTSD. To assure higher recruitment for future trials, it is preferable that the investigator works full-time at the study site during the study, and possibly more presentations and conferences can be held to educate and remind the clinicians about an ongoing study. Also, choosing a site with a larger research department and with clinicians more experienced in biological interventions would be a benefit. Designing a multicenter study, performing a study on inpatients, or including patients that are not currently in inpatient/outpatient care are other ways to increase recruitment. Finding other ways to pre-screen patients through a research nurse/assistant or through a survey/internet platform, where all patients at the clinic are systematically pre-screened for eligibility would be optimal. To ease the burden of the intervention on patients and help with compliance, it is recommended to provide the patients with complete nutrition and carefully choose which data to collect, to minimise registrations by the patient. Using continuous glucose and ketone monitors in future trials will collect more data while making it easier for the patients and preferably this data is available in real-time through an app or online platform, for both the patient and investigator, to be able to intervene fast and adjust the diet according to the results of measurements. Using continuous monitors may also make it feasible for patients not wanting to perform finger-prick blood measurements, e.g., PTSD patients who experienced bodily harm and torture, to participate. Using more simple interventions like exogenous ketone- or medium-chain triglycerides (MCT) supplementation with less restrictive diet protocols, is also something that should be examined in future trials. Exogenous supplemental lactate is of interest in brain injury research as it can boost cerebral energy metabolism and potentially reduce neurodegenerative processes (38, 154, 155) and could potentially be investigated in PTSD. To be able to compare the results of future studies, it is important to standardise ketogenic nutrition protocols both in animal and human studies.

## Data Availability

The original contributions presented in the study are included in the article/Supplementary material, further inquiries can be directed to the corresponding authors.
